# The gut microbiota in larvae of the housefly *Musca domestica* and their horizontal transfer through feeding

**DOI:** 10.1186/s13568-017-0445-7

**Published:** 2017-07-10

**Authors:** Yao Zhao, Wanqiang Wang, Fen Zhu, Xiaoyun Wang, Xiaoping Wang, Chaoliang Lei

**Affiliations:** 1 Hubei International Cooperation Base for Waste Conversion by Insects, Huazhong Agricultural University, No. 1 Shizishan Street, Hongshan District, Wuhan, 430070 China; 2 Hubei Insect Resources Utilization and Sustainable Pest Management Key Laboratory, Huazhong Agricultural University, Wuhan, 430070 China

**Keywords:** Housefly, Gut microbiota, Transferring, Wheat bran

## Abstract

House fly larvae provide a prolific and sustainable source of proteins used in poultry and fish feed. Wheat bran is a superior diet for house fly larvae and has been widely investigated to exploit its potential in the food and feed area. Using Illumina MiSeq 16S rDNA sequencing, this study investigated the gut microbiota of house fly larvae feeding on wheat bran and the bacterial community in the wheat bran. The bacterial communities in the house fly larvae were dominated by the phyla *Proteobacteria* and *Firmicutes*. *Enterobacteriaceae* and *Providencia* were the predominant bacteria at the family and genus levels, respectively. Some bacteria in the phyla *Actinobacteria*, *Proteobacteria*, *Bacteroidetes* and *Firmicutes* may be transferred from the gut of house flies to the wheat bran during feeding and may be involved in degrading and utilizing polysaccharides in the cell wall of wheat bran. The significance of the gut microbiota of house fly larvae, their transferring and roles in degradation of wheat bran is discussed. These findings regarding the gut microbiota of house fly larvae will provide opportunities for research on the impact of microbial communities on poultry and fish.

**Electronic supplementary material:**

The online version of this article (doi:10.1186/s13568-017-0445-7) contains supplementary material, which is available to authorized users.

## Introduction

The house fly, *Musca domestica*, is a cosmopolitan and synanthropic insect that serves as a vector for many human diseases (Gupta et al. 2012). However, the larvae are also resource insects with important potential applications. For instance, the larvae could be used in swine manure bioconversion and pollution control (Zhang et al. 2014). The larvae also have medicinal purposes, including beneficial effects on wounds, such as debridement (Wollina et al. 2000). The larvae also represent a sustainable and prolific source of proteins used in poultry and fish feed (Van 2013).

Because large volumes are required to supplement commercial poultry diets, the rearing technology for fly larvae requires further development. House flies can reproduce and develop in poultry and pig manure (Akpodiete et al. 1997; Zhu et al. 2012), but there are still a number of challenges to be addressed, including safety issues related to pathogens, heavy metals, and organic pollutants (Van 2013).

Wheat bran, the most important milling by-product of cereal grain (Prückler et al. 2014) and source of dietary fibre, minerals, vitamins and phenolic acids (Coda et al. 2013), is a superior diet for house fly larvae (Aniebo et al. 2008; Su et al. 2010). Many studies have investigated the bacterial community of adult house flies, which are considered pathogen vectors (Grübel et al. 1998; Gupta et al. 2012), but the gut microbiota in larvae and their transfer through food chain has not been characterized.

Early studies of bacterial diversity were primarily based on cultivation methods (Grübel et al. 1998; Zurek et al. 2000). However, many bacteria are uncultivable (Eilers et al. 2000). High-throughput DNA sequencing approaches provide a new means of characterizing bacterial communities and identifying cultivable and non-cultivable bacteria to provide an expanded perspective on bacterial diversity with higher coverage and a focus on a different set of organisms (Caporaso et al. 2010; Lozupone and Knight 2006). In this study, we used Illumina MiSeq 16S rDNA sequencing to identify the microbial dynamics of the gut microbiota in house fly larvae and their food. We are interested in (1) the microbial dynamics of the gut microbiota in house fly larvae and (2) their horizontal transfer through feeding.

## Materials and methods

### Sample collection

The house fly colony has been reared for more than 20 years in our lab. The house fly adults were fed with milk powder and water, and the larvae were reared on moistened wheat bran [wheat bran (g):water (ml) = 1:1.8]. In this experiment, newly hatched house fly eggs were inoculated into moistened wheat bran. After 2, 24, 48, 72 and 96 h, the house fly larvae were sampled (hereinafter referred to as Md02h, Md24h, Md48h, Md72h and Md96h). Moistened wheat bran treated with house fly larvae for 96 h was also sampled (hereinafter referred to as WBMd96h). As a control, moistened wheat bran not treated with house fly larvae was sampled after 24, 48, 72 and 96 h (hereinafter referred to as WB24h, WB48h, WB72h and WB96h). The experimental conditions were 28 ± 1 °C, 80 ± 5% relative humidity (RH), and a 13:11 h light:dark photoperiod (L:D). Three biological replicates were performed for each treatment.

### DNA extraction

Prior to insect dissection, the house fly larvae were washed for 3–5 min in 70% ethanol and rinsed three times with sterile water to remove surface contaminants. Each sample comprised three biological replicates, and each replicate contained 30 whole bodies of the 2 and 24-h larvae or 15 whole guts (from proventriculus to rectum, excluding Malpighian tubules) of the 48, 72 and 96-h larvae. The samples were then manually homogenized in extraction buffer (20 mM Tris–HCl pH 8.0, 2 mM sodium EDTA, 1.2% Triton^®^ X-100 containing 20 mg lysozyme ml^−1^). The homogenates were incubated at 37 °C for 1 h to extract DNA from both Gram-positive and Gram-negative bacteria. The DNA in the samples was then extracted using the TIANamp Genomic DNA Kit [TIANGEN Biotech (Beijing) LTD., China] following the manufacturer’s instructions. For wheat bran, 200 mg of each sample was used for DNA extraction with the TIANamp Stool DNA Kit [TIANGEN Biotech (Beijing) LTD., China], following the manufacturer’s instructions. The quantity and quality of the DNA were measured using a NanoDrop2000 spectrophotometer (Thermo Scientific, USA). DNA samples were stored at −80 °C until further processing.

### PCR amplification, library preparation and high-throughput sequencing

DNA was amplified using the 515f/806r primer set (515f: 5′-GTG CCA GCM GCC GCG GTA A-3′, 806r: 5′-XXX XXX GGA CTA CHV GGG TWT CTA AT-3′), which targets the V4 region of the bacterial 16S rDNA. The reverse primer contains a 6-bp error-correcting barcode unique to each sample. PCR amplifications were performed in a 30-μl mixture containing 15 μl of Phusion High-Fidelity PCR Master Mix (New England Biolabs, UK), 0.2 μM forward and reverse primers, 10 ng of template DNA and nuclease-free water up to 30 μl. The PCR conditions were 98 °C for 1 min (1 cycle), then 98 °C for 10 s, 50 °C for 30 s and 72 °C for 60 s (30 cycles), followed by 72 °C for 5 min. The PCR products were verified by 2% agarose gel electrophoresis and mixed in equidense ratios. The mixture of PCR products was purified using a GeneJET Gel Extraction Kit (Thermo Scientific, USA). Sequencing libraries were generated using a NEB Next Ultra DNA Library Prep Kit for Illumina (New England Biolabs, UK). Sequencing was conducted on an Illumina MiSeq 2 × 250 platform at BGI, Inc. (Shenzhen, China) according to protocols described by Caporaso et al. (2012) and Kozich et al. (2013).

### Bioinformatics and statistical analysis

Paired-end reads were assigned to samples based on their unique barcodes and truncated by cutting off the barcode and primer sequence. Then, the paired-end reads were merged into longer single sequences using FLASH (v1.2.11) (Magoč and Salzberg 2011). Quality filtering was performed on the raw tags under specific filtering conditions to obtain high-quality clean tags (Bokulich et al. 2013) according to the QIIME (v1.8.0) (Caporaso et al. 2010) quality-control process.

OTUs were clustered with a 97% similarity cut-off using UPARSE (v7.0.1090) (Edgar 2013). Chimeric sequences were detected and removed using UCHIME (v4.2.40) (Edgar et al. 2011). Representative sequences from each OTU were screened for further annotation. For each representative sequence, the GreenGene Database (DeSantis et al. 2006) was used with the RDP classifier (v2.2) (Wang et al. 2007) to annotate taxonomic information. Microbial diversity was analysed using QIIME v1.8.0 and displayed using R software (v3.0.3) (Caporaso et al. 2010). The alpha diversity analysis included observed species, Ace and Chao1 estimators, and the Simpson and Shannon diversity indices. The sequencing data have been submitted to the NCBI database under accession numbers SRP068683 and SRP068753.

## Results

### Sequencing data

The Illumina MiSeq sequencing of the 16S rRNA gene amplicons yielded 81,523–90,132 reads of house fly larvae samples and 77,843–83,590 reads of wheat bran samples, after quality filtering and the removal of chimeric sequences (Table 1). At 97% sequence identity, the reads for the house fly samples and wheat bran samples were assigned to 145 and 231 OTUs, respectively (Additional file 1: Tables S1, S2). The rarefaction curve for every sample tended to saturation (Additional file 1: Figure S1), indicating that our sequencing results captured most of the bacterial diversity.

**Table 1:** Richness and diversity estimates of the 16S rRNA gene libraries from the sequencing analysis

Sample	Tag number^a^	OTU number^b^	Ace	Chao1	Shannon	Simpson
Md02h	90132	83	96.29	96.57	1.35	0.30
Md24h	89186	68	81.33	81.33	0.81	0.56
Md48h	85270	77	80.98	79.55	1.79	0.28
Md72h	81523	92	106.81	113.00	2.33	0.14
Md96h	82378	98	107.94	106.27	1.94	0.22
WB24h	83590	85	130.94	113.88	2.28	0.13
WB48h	80037	90	97.75	99.17	2.50	0.12
WB72h	80319	112	115.05	113.50	2.69	0.09
WB96h	77843	116	123.94	121.63	2.91	0.09
WBMd96h	80089	165	170.33	167.50	3.08	0.07

MD02h, MD24h, MD48h, MD72h and MD96h refer to *Musca domestica* larvae reared on moistened wheat bran for 2, 24, 48, 72 and 96 h. WB24h, WB48h, WB72h and WB96h refer to moistened wheat bran not treated with house fly larvae after 24, 48, 72 and 96 h. WBMd96h refers to moistened wheat bran treated with house fly larvae for 96 h. Each treatment included three biological replicates

^a^Tag number after quality filtering and removal of chimeric sequences

^b^Operational taxonomic units (OTUs) were defined by pairwise 97% sequence identity

### Bacterial diversity in house fly larvae

**Fig. 1:**
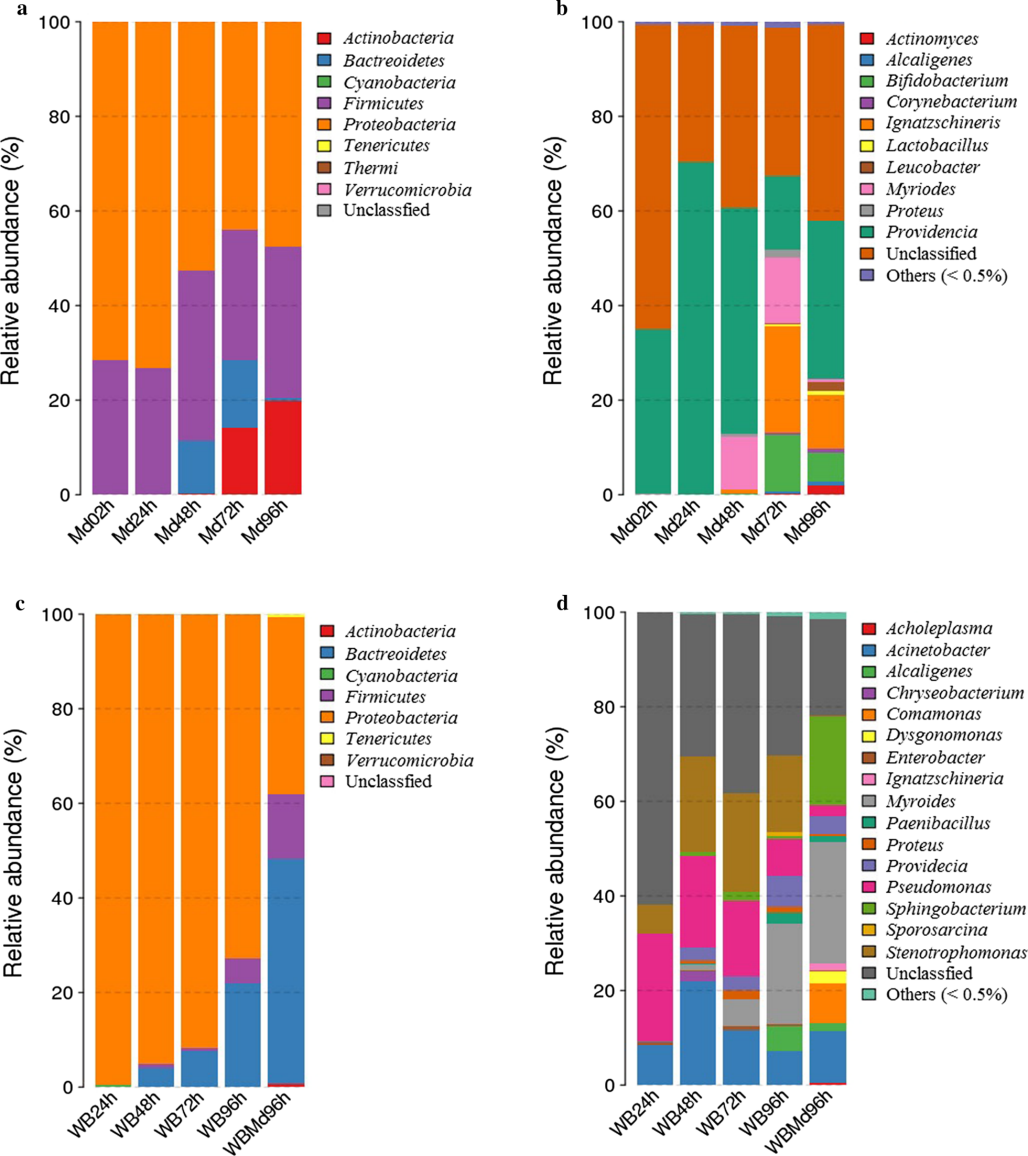
Relative abundances of bacteria at the phylum and genus levels in samples of *Musca domestica* larvae and wheat bran. **a** Relative abundances of bacteria at the phylum level in *M. domestica* larvae. **b** Relative abundances of bacteria at the genus level in *M. domestica* larvae. **c** Relative abundances of bacteria at the phylum level in wheat bran. **d** Relative abundances of bacteria at the genus level in wheat bran. MD02h, MD24h, MD48h, MD72h and MD96h refer to *Musca domestica* larvae reared on moistened wheat bran for 2, 24, 48, 72 and 96 h. WB24h, WB48h, WB72h and WB96h refer to moistened wheat bran not treated with house fly larvae after 24, 48, 72 and 96 h. WBMd96h refers to moistened wheat bran treated with house fly larvae for 96 h. Each treatment included three biological replicates

**Fig. 2:**
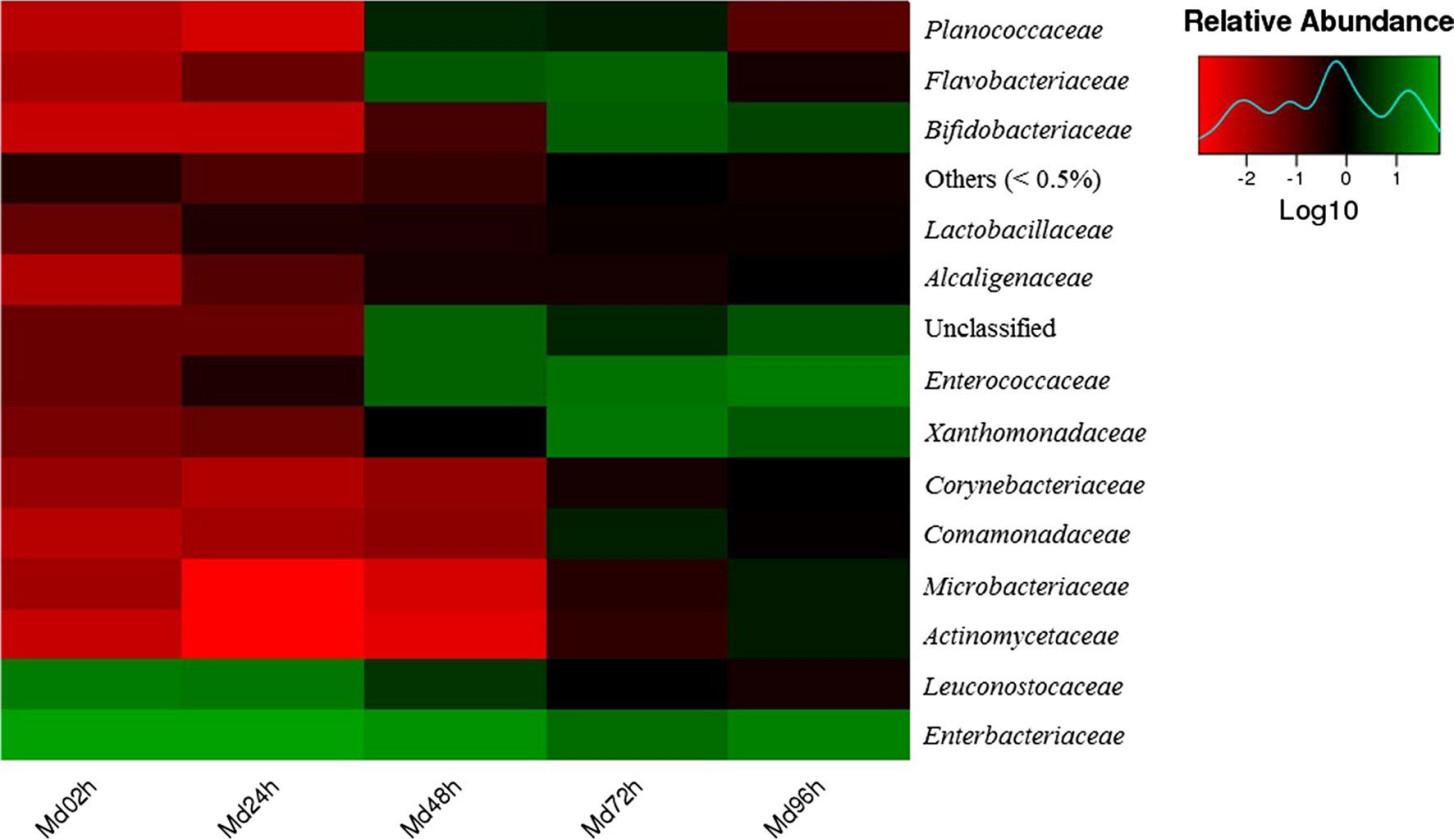
Heat maps of the relative abundances and distributions of bacterial families in *Musca domestica* larvae. The *colour code* indicates relative abundance, ranging from *red* (low abundance) to *black to green* (high abundance). Each treatment included three biological replicates. To minimize the degree of difference in relative abundance values, all values were log transformed

The bacterial communities in the house fly larvae samples were dominated by the phyla *Proteobacteria* and *Firmicutes* (Fig. 1a). The relative abundance of the phylum *Actinobacteria* was much higher in Md72h and Md96h samples than in the other three *M. domestica* samples (Fig. 1a). At the family level, *Enterobacteriaceae* was most dominant, with a relative abundance of nearly 50% (average value across all samples) (Fig. 2). *Providencia* dominated the bacterial communities at the genus level, with a relative abundance of 40.31% (Fig. 1b). Additionally, the Md72h and Md96h samples had generally higher Ace and Chao1 richness estimates compared with the samples Md02h, Md24h and Md48h (Table 1).

### Bacterial diversity in wheat bran

The bacterial communities in the control wheat bran samples (WB24h, WB48h, WB72h and WB96h) were dominated by the phylum *Proteobacteria*, and its relative abundance was nearly 90% (Fig. 1c). In the WBMd96h samples, the dominant phyla were *Proteobacteria* and *Bacteroidetes*, with relative abundances of 37.40 and 47.58%, respectively (Fig. 1c). At the genus level, *Myroides* and *Stenotrophomonas* were the major taxa in the control wheat bran samples (Fig. 1d). *Myroides* and *Sphingobacterium* were the major taxa in the WBMd96h samples (Fig. 1d). The relative abundance of the genus *Comamonas* was much higher in the WBMd96h samples compared with the WB96h samples (Fig. 1d).

**Fig. 3:**
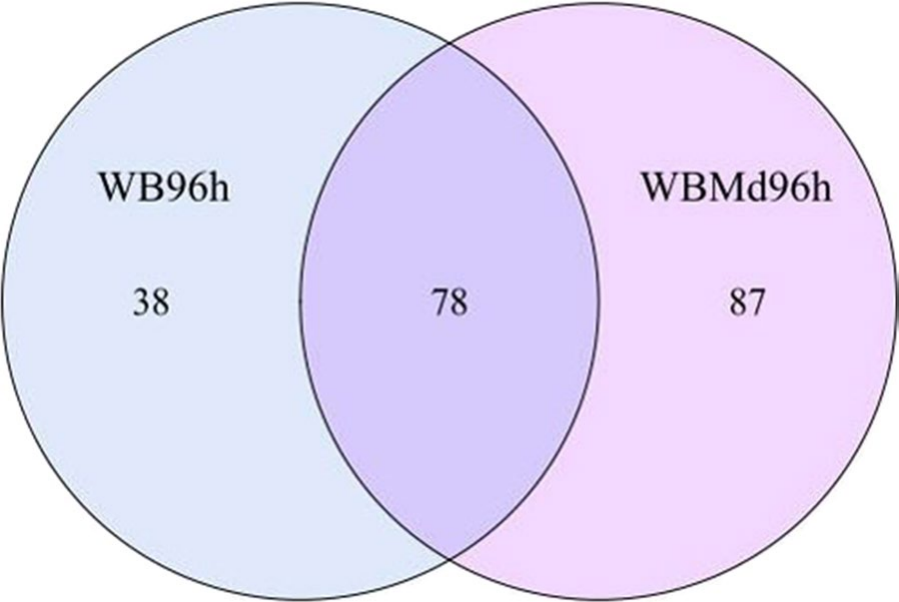
Venn diagram of WB96h and WBMd96h samples at distance 0.03. The *numbers* represent the number of unique OTUs in each sample and common OTUs shared by the two samples. WB96h refers to the moistened wheat bran not treated with house fly larvae after 96 h. WBMd96h refers to the moistened wheat bran treated with house fly larvae for 96 h. Each treatment included three biological replicates

The Venn diagram of the WB96h and WBMd96h samples revealed that 78 OTUs were shared by the two samples (Fig. 3). *Myroides* and *Acinetobacter* were the major genera in these common OTUs (Fig. 1d). There were 87 unique OTUs in the WBMd96h samples (Fig. 3), and *Dysgonomonas* was the major genus (Fig. 1d; Additional file 1: Table S3). Moreover, the WBMd96h samples had generally higher Ace and Chao1 richness estimates than the WB96h samples (Table 1).

## Discussion

To our knowledge, this study is the first to investigate the microbial dynamics of the gut microbiota in house fly larvae and their horizontal transfer through feeding. The bacterial communities in the house fly larvae samples were dominated by the phyla *Proteobacteria* and *Firmicutes*. The relative abundance of the phylum *Actinobacteria* was much higher in the Md72h and Md96h samples than in other house fly larvae samples. *Enterobacteriaceae* and *Providencia* were the predominant bacteria at the family and genus levels, respectively. Some bacteria in the phyla *Actinobacteria*, *Proteobacteria*, *Bacteroidetes* and *Firmicutes* were either unique to the WBMd96h samples or had much higher abundances in the WBMd96h samples compared with the WB96h samples, suggesting that they might have been transferred from the gut of the house fly to the wheat bran during feeding and might be involved in degrading and utilizing polysaccharides in the cell walls of wheat bran.

The bacterial communities in the guts of house fly larvae were dominated by the phylum *Proteobacteria* and primarily the class *Gammaproteobacteria* (Additional file 1: Table S1). *Gammaproteobacteria* are also commonly present in the guts of many other insects, such as the fruit fly *Drosophila melanogaster* (Corbyharris et al. 2007), the mosquito *Culex quinquefasciatus* (Pidiyar et al. 2004), the pea aphid *Acyrthosiphon pisum* (Oliver et al. 2010), the honeybee *Apis mellifera* (Jeyaprakash et al. 2003) and the gypsy moth *Lymantria dispar* (Broderick et al. 2004). Within *Proteobacteria*, members of the family *Enterobacteriaceae* dominated the bacterial communities, consistent with previous findings in the gut of house flies (Gupta et al. 2012). *Enterobacteriaceae* is also dominant in the gut of the flesh fly (Gupta et al. 2014) and some fruit fly species (Aharon et al. 2013; Behar et al. 2008; Wang et al. 2014). *Enterobacteriaceae* is a type of diazotrophic bacteria, which can help insects fix nitrogen (Dixon and Kahn 2004). Moreover, it has been reported that the *Enterobacteriaceae* community in the gut of medfly may indirectly contribute to host fitness by preventing the establishment or proliferation of pathogenic bacteria (Dillon and Dillon 2004).

*Firmicutes* was also a major component in the gut of house fly larvae. *Staphylococcus* belongs to this phylum (Additional file 1: Table S1) and has been frequently detected in other studies on house flies (Grübel et al. 1998; Gupta et al. 2012; Zurek et al. 2000). In the present study, *Actinobacteria* was another major phylum in the Md72h and Md96h samples, and the relative abundance of *Actinobacteria* was much higher in the WBMd96h than the WB96h samples. This result suggests that *Actinobacteria* transferred to the wheat bran when the house fly larvae were feeding. *Actinobacteria* associated with termites facilitate nutrient acquisition from diverse polysaccharides, including cellulose (Pasti and Belli 1985; Watanabe et al. 2003) and hemicelluloses (Schäfer et al. 1996), and *Actinobacteria* may similarly facilitate the utilization of polysaccharides in wheat bran by house flies. Arabinoxylans and β-glucans are polysaccharides in the cell wall of wheat bran and have a potential role in lowering the risk of type II diabetes, colorectal cancer and cardiovascular and diverticular diseases (Poutanen et al. 2014). *Actinobacteria* has also been reported to exhibit diverse physiological and metabolic properties, such as the production of extracellular enzymes and the formation of a wide variety of secondary metabolites (Schrempf 2001).

Although the gut microbiota of house flies growing in different habitats and on different diets vary, *Providencia* and *Proteus* are always present within the gut of house flies. For example, species of *Providencia* and *Proteus* were detected in the guts of laboratory-reared newly emerged adults (Su et al. 2010). Bacteria collected from adult house flies in public places also included the genera *Providencia* and *Proteus* (Gupta et al. 2012). Zurek et al. isolated *Providencia rettgeri* and *Providencia stuartii* from the intestinal tracts of house fly larvae collected from corn silage and turkey bedding (Zurek et al. 2000). In addition, Grubel et al. reported several bacterial species from the digestive tracts of laboratory-reared adult house flies, including *Providencia* (Grübel et al. 1998).

*Providencia* and *Proteus* were also detected in house fly larvae samples in the present study*. Providencia* is a genus of ubiquitous Gram-negative bacteria in the family *Enterobacteriaceae* and cause several human diseases (Gupta et al. 2012). *Providencia* have been identified as part of the normal human gut flora, and the genomes of some strains have been sequenced as part of the Human Microbiome Project (Stefano 2009). In addition, *Providencia* has been associated with numerous animals, including penguin (Muller 1983), sea turtles (Foti et al. 2009), shark (Interaminense et al. 2010), nematodes (Jackson et al. 1995) and snakes (Jho et al. 2011). *Providencia* strains have also been observed in association with various species of fly such as blowflies (Ahmad et al. 2006), stable flies (Mramba et al. 2006) and fruit flies (Aharon et al. 2013; Chandler et al. 2011; Corbyharris et al. 2007). For instance, *Providencia* strains have been isolated as infectious agents with varied virulence towards *D. melanogaster* ((Galac and Lazzaro 2011; Juneja and Lazzaro 2009). Additionally, some specific strains of *Providencia* can metabolize rhamnose (Galac and Lazzaro 2012). *Proteus* has been reported to protect the host from invasion by pathogenic microorganisms (Erdmann 1987; Greenberg and Klowden 1973). Greenberg and Klowden demonstrated that *Proteus mirabilis* is maintained at high levels in the gut of house fly larvae while suppressing the growth of two pathogenic microorganisms, *Salmonella typhimurium* and *Pseudomonas aeruginosa* (Greenberg and Klowden 1973). Erdmann determined that aromatic metabolites of *P. mirabilis* are involved in the suppression of pathogens in calliphorid larvae (Erdmann 1987). *P. mirabilis* from the salivary glands of the blow fly *Lucilia sericata* swarm significantly and produce a strong odour that attracts additional blow flies (Ma et al. 2012). We speculate that the genus *Proteus* may produce volatiles that serve as an oviposition attractant for the house fly.

It is well known that endosymbionts can confer ecologically relevant traits to their host. Symbiotic bacteria contributed to fitness of olive flies *Bactrocera oleae* (Ben-Yosef et al. 2010), and enable *B. oleae* to exploit intractable sources of nitrogen and overcome host defences (Ben-Yosef et al. 2014, 2015; Pavlidi et al. 2017). Endosymbionts could improve sterile male performance in Mediterranean fruit fly *Ceratitis capitata* (Yuval et al. 2013). In addition, substrate bacteria is also essential for larval survival and development (Zurek et al. 2000). The larvae of the stable fly *Stomoxys calcitrans* fail to develop on egg yolk medium not inoculated with bacteria but complete development on medium inoculated with *Acinetobacter* sp., *Empedobacter breve* and *Flavobacterium odoratum*, confirming that bacteria are required to complete development (Lysyk et al. 1999). The genera *Bacillus*, *Enterobacter* and *Myroides* were detected in our wheat bran samples (Additional file 1: Table S2), and specific species of these genera contribute to the development of *M. domestica* larvae (Su et al. 2010).

Apart from the bacteria in the phylum *Actinobacteria* discussed above, several other phyla were observed that might be involved in degrading and utilizing polysaccharides in the cell wall of wheat bran, such as *Proteobacteria*, *Bacteroidetes* and *Firmicutes*, including the family *Sphingobacteriaceae* and the genera *Comamonas*, *Dysgonomonas*, *Bacteroides*, *Lysinibacillus* and *Lactobacillus*. Compared with the WB96h samples, these bacteria were either unique to the WBMd96h samples or had much higher abundances in the WBMd96h samples, suggesting that these bacteria were transferred from the gut of the house fly to the wheat bran during feeding. Species of the family *Sphingobacteriaceae* are capable of degrading pectin, xylan, laminarin and other polysaccharides (Pankratov et al. 2007). The genus *Comamonas* can be used in the utilization and bioconversion of lignin (Chen et al. 2012). Furthermore, a microbial community including the genera *Dysgonomonas*, *Bacteroides* and *Lysinibacillus* expressed alkaliphilic xylanase, which may have potential implications in the pulp and paper industries (Lv et al. 2008). In addition, bioprocessing by *Lactobacillus*, yeast and cell-wall-degrading enzymes strongly increases the digestibility of proteins and phytase activity in wheat bran (Arte et al. 2015). The genera *Comamonas*, *Dysgonomonas* and *Bacteroides* were also detected in wild-collected house flies (Gupta et al. 2012; Wei et al. 2013), suggesting these genera may widely exist in the house fly. Further detailed studies of the bacteria identified in the present study may reveal potential applications in wheat bran processing and many other related areas.

Several other genera reported in the house fly (Grübel et al. 1998; Gupta et al. 2012; Zurek et al. 2000), such as *Serratia* and *Morganella*, were not detected in our study. This discrepancy may be attributable to differences in habitat, diet, life stage, etc. The bacterial diversity associated with *Anopheles gambiae* varies depending on the habitat of the mosquito (Wang et al. 2011). Bacterial abundances and distribution were found different between laboratory-reared flies and wild-collected flies (Aharon et al. 2013). Gut microbial communities and dominant taxa vary as a result of the influence of larval diet and nutrition (Broderick et al. 2004; Chandler et al. 2011). In addition, the diversity of bacteria occupying *Bactrocera dorsalis* vary across different life stages of the fly (Andongma et al. 2015). The sterilizing irradiation affected the gut bacterial community structure of the Mediterranean fruit fly *C. capitata* (Ami et al. 2010).House fly larvae may be a sustainable protein source, and the gut microbiota of these larvae represents an intriguing area of study for microbial ecology that will provide opportunities for research on the impact of microbial communities on poultry and fish. The findings presented here will also facilitate the elucidation of the roles of these bacteria in degrading and utilizing polysaccharides in the cell wall of wheat bran. Innovative and simple transformation processes will be critical to exploiting the nutritional quality of wheat bran and will also be applicable to industrial production.

## Additional file

**Additional file 1.** Additional figure and tables.
