# Supplementary material for: The gut microbiota in larvae of the housefly Musca domestica and their horizontal transfer through feeding
Source: AMB Express. 2017 Jul 10;7:147. doi: 10.1186/s13568-017-0445-7 (PMC5503848; doi:10.1186/s13568-017-0445-7)
Supplement: Supplementary file 1 — Additional file 1. Additional figure and tables. [file 13568_2017_445_MOESM1_ESM.pdf]

Supplementary Information

The gut microbiota in larvae of the housefly *Musca domestica* and their horizontal transfer through feeding

AMB Express

Yao Zhao[1], Wanqiang Wang[1], Fen Zhu[1], Xiaoyun Wang[1], Xiaoping Wang[2], Chaoliang Lei[2]

[1]Hubei International Cooperation Base for Waste Conversion by Insects, Huazhong Agricultural University, Wuhan 430070, China; [2]Hubei Insect Resources Utilization and Sustainable Pest Management Key Laboratory, Huazhong Agricultural University, Wuhan 430070, China.

Correspondence: F Zhu, Hubei International Cooperation Base for Waste Conversion by Insects, Huazhong Agricultural University, No. 1 Shizishan Street, Wuhan 430070, China

E-mail: zhufen@mail.hzau.edu.cn

This Supplementary Information contains:

Supplementary Figure Legends

Supplementary Figures S1

Supplementary Tables S1-S3

Supplementary Figure Legends

Figure S1. Rarefaction curve based on bacterial OTUs at a dissimilarity level of 3%. (A) Rarefaction curve of *Musca domestica* samples. (B) Rarefaction curve of wheat bran samples. MD02h, MD24h, MD48h, MD72h and MD96h refer to *Musca domestica* larvae reared on moistened wheat bran for 2, 24, 48, 72 and 96 h. WB24h, WB48h, WB72h and WB96h refer to moistened wheat bran not treated with housefly larvae after 24, 48, 72 and 96 h. WBMd96h refers to moistened wheat bran treated with housefly larvae for 96 h. Each treatment included three biological replicates.

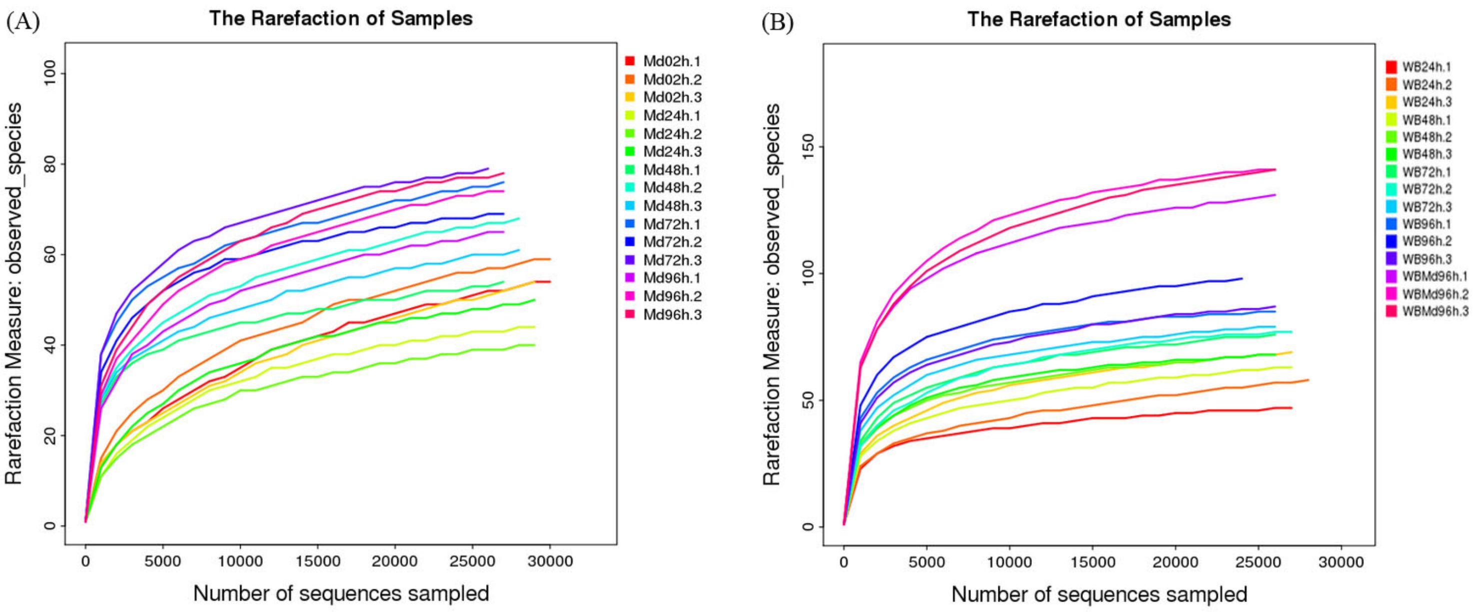
(A)
The Rarefaction of Samples
Rarefaction Measure: observed_species
Number of sequences sampled
0
20
40
60
80
100
0
5000
10000
15000
20000
25000
30000
Md02h.1
Md02h.2
Md02h.3
Md24h.1
Md24h.2
Md24h.3
Md48h.1
Md48h.2
Md48h.3
Md72h.1
Md72h.2
Md72h.3
Md96h.1
Md96h.2
Md96h.3
(B)
The Rarefaction of Samples
Rarefaction Measure: observed_species
Number of sequences sampled
0
50
100
150
0
5000
10000
15000
20000
25000
30000
WB24h.1
WB24h.2
WB24h.3
WB48h.1
WB48h.2
WB48h.3
WB72h.1
WB72h.2
WB72h.3
WB96h.1
WB96h.2
WB96h.3
WBMd96h.1
WBMd96h.2
WBMd96h.3

Fig. S1

Supplementary Table S1. Bacterial taxonomy and abundance in the gut of *Musca domestica* larvae at different developmental stages.

OTU	Md02h	Md24h	Md48h	Md72h	Md96h	Taxonomy
OTU1	31290	62579	40799	12609	27495	*Bacteria; Proteobacteria; Gammaproteobacteria; Enterobacteriales; Enterobacteriaceae; Providencia*
OTU2	25332	23075	3582	852	445	*Bacteria; Firmicutes; Bacilli; Lactobacillales; Leuconostocaceae*
OTU3	28619	1678	1652	420	12	*Bacteria; Proteobacteria; Gammaproteobacteria; Enterobacteriales; Enterobacteriaceae*
OTU4	5	42	8678	6108	214	*Bacteria; Bacteroidetes; Flavobacteriia; Flavobacteriales; Flavobacteriaceae; Myroides*
OTU5	160	23	4	5	9	*Bacteria; Proteobacteria; Gammaproteobacteria; Pseudomonadales; Moraxellaceae; Acinetobacter*
OTU6	7	18	671	18222	9368	*Bacteria; Proteobacteria; Gammaproteobacteria; Xanthomonadales; Xanthomonadaceae; Ignatzschineria*
OTU7	24	9	4	3	1	*Bacteria; Proteobacteria; Gammaproteobacteria; Pseudomonadales; Pseudomonadaceae; Pseudomonas; Pseudomonas_pseudoalcaligenes*
OTU8	50	347	11936	17226	24004	*Bacteria; Firmicutes; Bacilli; Lactobacillales; Enterococcaceae*
OTU9	6	10	11165	859	773	*Bacteria; Firmicutes; Bacilli; Lactobacillales*
OTU10	5	5	136	9772	4916	*Bacteria; Actinobacteria; Actinobacteria; Bifidobacteriales; Bifidobacteriaceae; Bifidobacterium*
OTU11	6	10	784	5098	208	*Bacteria; Bacteroidetes; Flavobacteriia; Flavobacteriales; Flavobacteriaceae; Myroides*
OTU12	5	8	9	776	7153	*Bacteria; Actinobacteria; Actinobacteria; Actinomycetales*
OTU13	16	17	0	0	0	*Bacteria; Proteobacteria; Gammaproteobacteria; Xanthomonadales; Xanthomonadaceae; Stenotrophomonas*
OTU14	2	0	2	12	51	*Bacteria; Firmicutes; Bacilli; Bacillales; Bacillaceae*
OTU15	6	5	1	4	17	*Bacteria; Bacteroidetes; Sphingobacteriia; Sphingobacteriales; Sphingobacteriaceae; Sphingobacterium; Sphingobacterium_multivorum*
OTU16	0	3	5	33	9	*Bacteria; Proteobacteria; Betaproteobacteria; Burkholderiales; Comamonadaceae; Comamonas*
OTU17	1	0	2	49	18	*Bacteria; Proteobacteria; Betaproteobacteria; Burkholderiales; Alcaligenaceae; Alcaligenes; Alcaligenes_faecalis*
OTU19	2466	494	217	125	138	*Bacteria; Proteobacteria; Gammaproteobacteria; Enterobacteriales; Enterobacteriaceae*
OTU21	3	3	2398	1677	40	*Bacteria; Firmicutes; Bacilli; Bacillales; Planococcaceae*
OTU22	0	0	0	0	2	*Bacteria; Proteobacteria; Gammaproteobacteria; Pseudomonadales; Pseudomonadaceae; Pseudomonas*
OTU23	176	41	470	1356	22	*Bacteria; Proteobacteria; Gammaproteobacteria; Enterobacteriales; Enterobacteriaceae; Proteus*
OTU24	0	1	7	0	0	*Bacteria; Proteobacteria; Gammaproteobacteria; Pseudomonadales; Moraxellaceae; Acinetobacter*

OTU25	0	2	83	227	645	*Bacteria; Proteobacteria; Betaproteobacteria; Burkholderiales; Alcaligenaceae; Alcaligenes; Alcaligenes_faecalis*
OTU26	0	2	0	0	5	*Bacteria; Firmicutes; Bacilli; Bacillales; Paenibacillaceae; Paenibacillus*
OTU27	3	1	1	9	4	*Bacteria; Bacteroidetes; Bacteroidia; Bacteroidales; Porphyromonadaceae; Dysgonomonas*
OTU28	0	1	0	0	4	*Bacteria; Proteobacteria; Gammaproteobacteria; Pseudomonadales; Pseudomonadaceae; Pseudomonas*
OTU29	6	0	2	171	1327	*Bacteria; Actinobacteria; Actinobacteria; Actinomycetales; Microbacteriaceae; Leucobacter*
OTU31	0	0	0	0	11	*Bacteria; Bacteroidetes; Flavobacteriia; Flavobacteriales; Flavobacteriaceae; Myroides*
OTU32	5	1	2	237	1641	*Bacteria; Actinobacteria; Actinobacteria; Actinomycetales; Actinomycetaceae; Actinomyces*
OTU33	15	6	17	428	797	*Bacteria; Actinobacteria; Actinobacteria; Actinomycetales; Corynebacteriaceae; Corynebacterium*
OTU34	1	0	0	0	0	*Bacteria; Proteobacteria; Alphaproteobacteria; Rickettsiales; mitochondria*
OTU36	17	0	0	0	1	*Bacteria; Proteobacteria; Gammaproteobacteria; Pseudomonadales; Moraxellaceae; Acinetobacter; Acinetobacter_rhizosphaerae*
OTU37	0	0	0	3	0	*Bacteria; Firmicutes; Bacilli; Bacillales; Planococcaceae; Sporosarcina*
OTU38	0	1	4	357	583	*Bacteria; Firmicutes; Bacilli; Lactobacillales; Lactobacillaceae; Lactobacillus; Lactobacillus_reuteri*
OTU39	63	356	229	126	24	*Bacteria; Firmicutes; Bacilli; Lactobacillales; Lactobacillaceae; Pediococcus*
OTU40	2	1	99	233	135	*Bacteria; Proteobacteria; Alphaproteobacteria; Rhizobiales; Brucellaceae*
OTU41	6	0	0	1	0	*Bacteria; Cyanobacteria; Chloroplast; Streptophyta*
OTU42	5	74	299	120	110	*Bacteria; Proteobacteria; Betaproteobacteria; Burkholderiales; Alcaligenaceae; Achromobacter*
OTU45	0	0	25	216	0	*Bacteria; Bacteroidetes; Bacteroidia; Bacteroidales; Bacteroidaceae; Bacteroides*
OTU46	0	0	146	348	6	*Bacteria; Proteobacteria; Gammaproteobacteria; Xanthomonadales; Xanthomonadaceae; Wohlfahrtiimonas*
OTU50	1	0	0	0	0	*Bacteria; Tenericutes; Mollicutes; Acholeplasmatales; Acholeplasmataceae; Acholeplasma*
OTU51	0	0	2	12	141	*Bacteria; Proteobacteria; Alphaproteobacteria; Rhizobiales; Xanthobacteraceae; Xanthobacter*
OTU53	3	0	0	1	2	*Bacteria; Actinobacteria; Actinobacteria; Actinomycetales*
OTU55	0	0	0	1	1	*Bacteria; Proteobacteria; Gammaproteobacteria; Pseudomonadales; Pseudomonadaceae; Pseudomonas; Pseudomonas_nitroreducens*
OTU58	0	0	1	35	34	*Bacteria; Proteobacteria; Alphaproteobacteria; Rhizobiales; Brucellaceae; Ochrobactrum*
OTU59	4	6	108	40	0	*Bacteria; Firmicutes; Bacilli; Lactobacillales; Lactobacillaceae*
OTU60	1	0	4	3	2	*Bacteria; Firmicutes; Bacilli; Bacillales; Planococcaceae; Lysinibacillus*

OTU62	2	0	5	117	19	*Bacteria; Firmicutes; Erysipelotrichi; Erysipelotrichales; Erysipelotrichaceae; Erysipelothrix*
OTU63	0	0	0	14	2	*Bacteria; Bacteroidetes; Bacteroidia; Bacteroidales; Porphyromonadaceae; Dysgonomonas*
OTU67	1	0	0	0	0	*Bacteria; Proteobacteria; Alphaproteobacteria; Sphingomonadales; Sphingomonadaceae; Novosphingobium*
OTU68	1	1	0	0	1	*Bacteria; Actinobacteria; Actinobacteria; Actinomycetales; Micrococcaceae*
OTU71	0	0	0	0	1	*Bacteria; Proteobacteria; Gammaproteobacteria; Xanthomonadales; Xanthomonadaceae; Stenotrophomonas; Stenotrophomonas_acidaminiphila*
OTU73	305	13	1	0	0	*Bacteria; Proteobacteria; Gammaproteobacteria; Enterobacteriales; Enterobacteriaceae*
OTU76	0	0	0	1	32	*Bacteria; Firmicutes; Bacilli; Lactobacillales; Aerococcaceae; Facklamia*
OTU80	0	0	0	2	10	*Bacteria; Proteobacteria; Betaproteobacteria; Burkholderiales; Alcaligenaceae; Oligella*
OTU82	1	8	17	50	3	*Bacteria; Firmicutes; Bacilli; Lactobacillales*
OTU85	43	8	2	1	5	*Bacteria; Firmicutes; Bacilli; Bacillales; Staphylococcaceae; Staphylococcus*
OTU86	0	0	6	30	0	*Bacteria; Proteobacteria; Gammaproteobacteria; Pasteurellales*
OTU88	1	11	4	1	1	*Bacteria; Thermi; Deinococci; Thermales; Thermaceae; Thermus*
OTU89	11	29	4	10	9	*Bacteria; Proteobacteria; Gammaproteobacteria; Xanthomonadales; Xanthomonadaceae*
OTU91	484	69	157	71	52	*Bacteria; Proteobacteria; Gammaproteobacteria; Enterobacteriales; Enterobacteriaceae*
OTU93	0	0	17	19	0	*Bacteria; Bacteroidetes; Flavobacteriia; Flavobacteriales; Flavobacteriaceae; Myroides; Myroides_odoratimimus*
OTU94	2	0	0	46	319	*Bacteria; Actinobacteria; Actinobacteria; Actinomycetales; Microbacteriaceae; Leucobacter*
OTU95	0	2	3	0	4	*Bacteria; Bacteroidetes; Sphingobacteriia; Sphingobacteriales; Sphingobacteriaceae; Sphingobacterium; Sphingobacterium_multivorum*
OTU97	1	0	0	0	0	*Bacteria; Bacteroidetes; Flavobacteriia; Flavobacteriales; Weeksellaceae; Wautersiella*
OTU99	6	15	4	9	0	*Bacteria; Thermi; Deinococci; Thermales; Thermaceae; Thermus*
OTU100	15	7	0	0	0	*Bacteria; Proteobacteria; Alphaproteobacteria; Rhodospirillales; Acetobacteraceae; Commensalibacter; Commensalibacter_intestini*
OTU105	0	0	0	2	34	*Bacteria; Actinobacteria; Actinobacteria; Actinomycetales; Dietziaceae; Dietzia*
OTU106	0	1	2	4	16	*Bacteria; Proteobacteria; Alphaproteobacteria; Rhizobiales; Brucellaceae*
OTU107	1	1	0	0	0	*Bacteria; Proteobacteria; Betaproteobacteria; Burkholderiales; Comamonadaceae; Verminephrobacter*
OTU109	7	23	194	501	262	*Bacteria; Firmicutes; Bacilli; Lactobacillales; Enterococcaceae*
OTU111	3	2	5	14	4	*Bacteria; Proteobacteria; Alphaproteobacteria; Rhizobiales; Brucellaceae*

OTU112	0	0	2	9	5	*Bacteria; Actinobacteria; Actinobacteria; Actinomycetales; Brevibacteriaceae; Brevibacterium*
OTU113	0	0	0	0	22	*Bacteria; Actinobacteria; Actinobacteria; Actinomycetales; Microbacteriaceae; Pseudoclavibacter; Pseudoclavibacter_bifida*
OTU114	0	0	0	9	0	*Bacteria; Firmicutes; Erysipelotrichi; Erysipelotrichales; Erysipelotrichaceae; Erysipelothrix*
OTU115	24	0	3	0	0	*Bacteria; Proteobacteria; Gammaproteobacteria; Enterobacteriales; Enterobacteriaceae*
OTU118	1	0	0	0	0	*Bacteria; Proteobacteria; Betaproteobacteria; Burkholderiales; Oxalobacteraceae*
OTU119	5	0	0	0	5	*Bacteria; Firmicutes; Bacilli; Bacillales*
OTU121	0	0	16	0	1	*Bacteria; Firmicutes; Bacilli; Lactobacillales; Lactobacillaceae; Lactobacillus; Lactobacillus_paralimentarius*
OTU124	1	0	0	0	0	*Bacteria; Proteobacteria; Gammaproteobacteria; Xanthomonadales; Xanthomonadaceae; Stenotrophomonas*
OTU125	0	0	0	2	0	*Bacteria; Proteobacteria; Epsilonproteobacteria; Campylobacterales; Campylobacteraceae; Arcobacter*
OTU126	3	0	1	0	6	*Bacteria; Proteobacteria; Gammaproteobacteria; Pseudomonadales; Moraxellaceae; Acinetobacter*
OTU128	0	1	178	12	1	*Bacteria; Firmicutes; Bacilli*
OTU129	2	0	4	53	28	*Bacteria; Firmicutes; Bacilli; Bacillales; Planococcaceae*
OTU133	0	0	3	7	8	*Bacteria; Firmicutes; Bacilli; Lactobacillales; Enterococcaceae; Enterococcus*
OTU134	0	1	0	0	3	*Bacteria; Proteobacteria; Gammaproteobacteria; Xanthomonadales; Xanthomonadaceae; Stenotrophomonas*
OTU140	0	0	0	1	1	*Bacteria; Proteobacteria; Betaproteobacteria; Burkholderiales*
OTU141	5	0	0	0	0	*Bacteria; Proteobacteria; Betaproteobacteria; Burkholderiales; Oxalobacteraceae*
OTU144	0	0	14	4	0	*Bacteria; Firmicutes; Bacilli; Lactobacillales; Carnobacteriaceae*
OTU145	0	0	0	4	0	*Bacteria; Proteobacteria; Gammaproteobacteria; Xanthomonadales; Xanthomonadaceae; Ignatzschineria*
OTU147	0	0	0	0	1	*Bacteria; Firmicutes; Bacilli; Bacillales; Bacillaceae*
OTU148	2	5	0	0	0	*Bacteria; Proteobacteria; Gammaproteobacteria; Enterobacteriales; Enterobacteriaceae*
OTU150	5	0	0	3	2	*Bacteria; Actinobacteria; Actinobacteria; Actinomycetales; Microbacteriaceae*
OTU151	1	2	0	3	2	*Bacteria; Proteobacteria; Betaproteobacteria; Burkholderiales; Comamonadaceae*
OTU152	14	1	0	1	0	*Bacteria; Proteobacteria; Gammaproteobacteria; Pseudomonadales; Moraxellaceae; Acinetobacter*
OTU157	0	1	0	0	0	*Bacteria; Proteobacteria; Gammaproteobacteria*
OTU161	0	0	0	0	2	*Bacteria; Firmicutes; Erysipelotrichi; Erysipelotrichales; Erysipelotrichaceae*

OTU162	2	18	60	65	131	*Bacteria; Proteobacteria; Betaproteobacteria; Burkholderiales; Alcaligenaceae*
OTU164	199	37	23	1	0	*Bacteria; Proteobacteria; Gammaproteobacteria; Enterobacteriales; Enterobacteriaceae; Enterobacter*
OTU165	0	0	0	43	3	*Bacteria; Firmicutes; Bacilli; Bacillales; Planococcaceae*
OTU166	1	7	92	43	9	*Bacteria; Firmicutes; Bacilli; Lactobacillales*
OTU167	0	0	67	105	9	*Bacteria; Bacteroidetes; Flavobacteriia; Flavobacteriales; Flavobacteriaceae; Myroides*
OTU169	0	0	0	0	5	*Bacteria; Actinobacteria; Actinobacteria; Actinomycetales; Microbacteriaceae; Leucobacter*
OTU171	0	2	14	9	21	*Bacteria; Firmicutes; Bacilli; Lactobacillales*
OTU172	1	0	0	0	0	*Bacteria; Proteobacteria; Gammaproteobacteria; Enterobacteriales; Enterobacteriaceae*
OTU177	1	5	4	0	0	*Bacteria; Firmicutes; Bacilli; Bacillales; Paenibacillaceae; Ammoniphilus*
OTU184	3	0	0	0	0	*Bacteria; Proteobacteria; Gammaproteobacteria; Enterobacteriales; Enterobacteriaceae*
OTU187	0	0	0	52	83	*Bacteria; Actinobacteria; Actinobacteria; Actinomycetales; Microbacteriaceae*
OTU188	2	0	0	0	0	*Bacteria; Proteobacteria; Gammaproteobacteria; Alteromonadales; Alteromonadaceae*
OTU189	0	0	0	2	0	*Bacteria; Actinobacteria; Actinobacteria*
OTU190	0	0	98	11	0	*Bacteria; Firmicutes; Bacilli*
OTU196	1	0	0	0	2	*Bacteria; Proteobacteria; Gammaproteobacteria; Enterobacteriales; Enterobacteriaceae*
OTU198	0	0	0	1	0	*Bacteria; Proteobacteria; Alphaproteobacteria; Rhizobiales*
OTU199	0	0	7	7	16	*Bacteria; Proteobacteria; Betaproteobacteria; Burkholderiales; Alcaligenaceae; Alcaligenes; Alcaligenes_faecalis*
OTU200	0	7	132	260	53	*Bacteria; Firmicutes; Bacilli; Lactobacillales*
OTU201	1	0	0	0	0	*Bacteria; Proteobacteria; Alphaproteobacteria; Rhodobacterales; Rhodobacteraceae; Paracoccus*
OTU205	0	1	0	0	9	*Bacteria; Actinobacteria; Actinobacteria; Actinomycetales; Microbacteriaceae; Microbacterium*
OTU209	0	0	1	35	50	*Bacteria; Actinobacteria; Actinobacteria; Actinomycetales; Microbacteriaceae*
OTU210	0	0	0	0	3	*Bacteria; Firmicutes; Bacilli; Bacillales*
OTU216	0	0	0	0	6	*Bacteria; Proteobacteria*
OTU219	4	1	0	1	0	*Bacteria; Proteobacteria; Alphaproteobacteria; Rhizobiales*
OTU220	1	0	0	0	0	*Bacteria; Proteobacteria; Alphaproteobacteria; Rickettsiales; mitochondria*

OTU221	6	6	0	0	0	*Bacteria; Firmicutes; Bacilli; Lactobacillales; Leuconostocaceae*
OTU222	0	12	47	89	18	*Bacteria; Firmicutes; Bacilli; Lactobacillales; Lactobacillaceae; Lactobacillus; Lactobacillus_brevis*
OTU223	0	0	0	0	3	*Bacteria; Proteobacteria; Alphaproteobacteria; Sphingomonadales; Sphingomonadaceae; Kaistobacter*
OTU224	2	1	0	1	4	*Bacteria; Proteobacteria; Betaproteobacteria; Burkholderiales; Comamonadaceae; Limnohabitans*
OTU227	0	0	62	3	0	*Bacteria; Firmicutes; Bacilli; Lactobacillales*
OTU230	2	0	0	0	0	*Bacteria; Proteobacteria; Deltaproteobacteria; Myxococcales*
OTU231	593	35	105	42	1	*Bacteria; Proteobacteria; Gammaproteobacteria; Enterobacteriales; Enterobacteriaceae*
OTU232	3	0	0	0	1	*Bacteria; Proteobacteria; Alphaproteobacteria; Caulobacterales; Caulobacteraceae*
OTU233	0	0	0	1	11	*Bacteria; Actinobacteria; Actinobacteria; Actinomycetales; Actinomycetaceae; Actinomyces*
OTU234	0	0	0	0	4	*Bacteria; Actinobacteria; Actinobacteria; Actinomycetales*
OTU235	2	0	0	1	0	*Bacteria; Bacteroidetes; Bacteroidia; Bacteroidales; S24-7*
OTU239	0	0	0	0	2	*Bacteria*
OTU242	0	8	1	0	2	*Bacteria; Firmicutes; Clostridia; Clostridiales; Lachnospiraceae*
OTU244	0	0	173	65	1	*Bacteria; Firmicutes; Bacilli; Lactobacillales*
OTU245	0	0	0	0	3	*Bacteria; Firmicutes; Bacilli; Bacillales*
OTU247	0	0	1	8	0	*Bacteria; Bacteroidetes; Bacteroidia; Bacteroidales; Porphyromonadaceae; Dysgonomonas*
OTU249	25	5	0	0	0	*Bacteria; Proteobacteria; Gammaproteobacteria; Pseudomonadales; Moraxellaceae; Acinetobacter*
OTU250	17	11	2	1	0	*Bacteria; Proteobacteria; Gammaproteobacteria*
OTU251	2	2	0	0	0	*Bacteria; Actinobacteria; Actinobacteria; Actinomycetales; Corynebacteriaceae; Corynebacterium*
OTU252	0	0	179	38	16	*Bacteria; Firmicutes; Bacilli; Lactobacillales*
OTU254	2	0	0	0	0	*Bacteria; Proteobacteria; Deltaproteobacteria*
OTU256	1	2	2	0	1	*Bacteria; Verrucomicrobia; Verrucomicrobiae; Verrucomicrobiales; Verrucomicrobiaceae; Akkermansia; Akkermansia_muciniphila*
OTU257	3	6	14	1865	682	*Bacteria; Proteobacteria; Betaproteobacteria; Burkholderiales; Comamonadaceae*

MD02h, MD24h, MD48h, MD72h and MD96h refer to *Musca domestica* larvae reared on moistened wheat bran for 2, 24, 48, 72 and 96 h. Each treatment included three biological replicates.

Supplementary Table S2. Bacterial taxonomy and abundance in wheat bran.

OTU	WB24h	WB48h	WB72h	WB96h	WBMd96h	Taxonomy
OTU1	120	2262	2370	5084	3050	*Bacteria; Proteobacteria; Gammaproteobacteria; Enterobacteriales; Enterobacteriaceae; Providencia*
OTU2	0	114	0	0	63	*Bacteria; Firmicutes; Bacilli; Lactobacillales; Leuconostocaceae*
OTU3	13564	11548	10951	9554	786	*Bacteria; Proteobacteria; Gammaproteobacteria; Enterobacteriales; Enterobacteriaceae*
OTU4	51	905	4452	14488	6837	*Bacteria; Bacteroidetes; Flavobacteriia; Flavobacteriales; Flavobacteriaceae; Myroides*
OTU5	6107	15921	8337	4090	3658	*Bacteria; Proteobacteria; Gammaproteobacteria; Pseudomonadales; Moraxellaceae; Acinetobacter*
OTU5	0	0	0	0	3	*Bacteria; Bacteroidetes; Bacteroidia; Bacteroidales; Porphyromonadaceae; Dysgonomonas*
OTU6	2	0	0	2	1157	*Bacteria; Proteobacteria; Gammaproteobacteria; Xanthomonadales; Xanthomonadaceae; Ignatzschineria*
OTU7	16848	14183	11684	5615	276	*Bacteria; Proteobacteria; Gammaproteobacteria; Pseudomonadales; Pseudomonadaceae; Pseudomonas; Pseudomonas_pseudoalcaligenes*
OTU8	3	47	24	9	1731	*Bacteria; Firmicutes; Bacilli; Lactobacillales; Enterococcaceae*
OTU9	0	0	0	0	12	*Bacteria; Firmicutes; Bacilli; Lactobacillales*
OTU10	0	0	0	0	16	*Bacteria; Actinobacteria; Actinobacteria; Bifidobacteriales; Bifidobacteriaceae; Bifidobacterium*
OTU11	0	0	58	1930	12329	*Bacteria; Bacteroidetes; Flavobacteriia; Flavobacteriales; Flavobacteriaceae; Myroides*
OTU12	0	0	0	0	61	*Bacteria; Actinobacteria; Actinobacteria; Actinomycetales*
OTU13	3897	11848	12498	9481	18	*Bacteria; Proteobacteria; Gammaproteobacteria; Xanthomonadales; Xanthomonadaceae; Stenotrophomonas*
OTU14	0	0	30	15	5685	*Bacteria; Firmicutes; Bacilli; Bacillales; Bacillaceae*
OTU15	1	388	746	158	8820	*Bacteria; Bacteroidetes; Sphingobacteriia; Sphingobacteriales; Sphingobacteriaceae; Sphingobacterium; Sphingobacterium_multivorum*
OTU16	1	9	4	22	6848	*Bacteria; Proteobacteria; Betaproteobacteria; Burkholderiales; Comamonadaceae; Comamonas*
OTU17	2	17	33	3116	435	*Bacteria; Proteobacteria; Betaproteobacteria; Burkholderiales; Alcaligenaceae; Alcaligenes; Alcaligenes_faecalis*
OTU18	0	1626	13	0	0	*Bacteria; Bacteroidetes; Flavobacteriia; Flavobacteriales; Weeksellaceae; Chryseobacterium*
OTU19	3444	3022	3612	3347	308	*Bacteria; Proteobacteria; Gammaproteobacteria; Enterobacteriales; Enterobacteriaceae*
OTU20	2085	103	330	12	1	*Bacteria; Proteobacteria; Gammaproteobacteria; Pseudomonadales; Pseudomonadaceae; Pseudomonas*
OTU21	0	0	0	223	258	*Bacteria; Firmicutes; Bacilli; Bacillales; Planococcaceae*

OTU22	50	1081	812	422	31	*Bacteria; Proteobacteria; Gammaproteobacteria; Pseudomonadales; Pseudomonadaceae; Pseudomonas*
OTU23	10	541	1551	926	453	*Bacteria; Proteobacteria; Gammaproteobacteria; Enterobacteriales; Enterobacteriaceae; Proteus*
OTU24	40	146	244	1076	66	*Bacteria; Proteobacteria; Gammaproteobacteria; Pseudomonadales; Moraxellaceae; Acinetobacter*
OTU25	10	4	12	968	762	*Bacteria; Proteobacteria; Betaproteobacteria; Burkholderiales; Alcaligenaceae; Alcaligenes; Alcaligenes_faecalis*
OTU26	1	3	17	1277	679	*Bacteria; Firmicutes; Bacilli; Bacillales; Paenibacillaceae; Paenibacillus*
OTU27	0	0	0	0	1913	*Bacteria; Bacteroidetes; Bacteroidia; Bacteroidales; Porphyromonadaceae; Dysgonomonas*
OTU28	0	0	0	0	1469	*Bacteria; Proteobacteria; Gammaproteobacteria; Pseudomonadales; Pseudomonadaceae; Pseudomonas*
OTU29	0	0	0	0	60	*Bacteria; Actinobacteria; Actinobacteria; Actinomycetales; Microbacteriaceae; Leucobacter*
OTU30	1	0	128	823	6	*Bacteria; Firmicutes; Bacilli; Bacillales; Bacillaceae*
OTU31	0	0	0	0	1179	*Bacteria; Bacteroidetes; Flavobacteriia; Flavobacteriales; Flavobacteriaceae; Myroides*
OTU32	0	0	0	0	75	*Bacteria; Actinobacteria; Actinobacteria; Actinomycetales; Actinomycetaceae; Actinomyces*
OTU33	0	0	0	0	8	*Bacteria; Actinobacteria; Actinobacteria; Actinomycetales; Corynebacteriaceae; Corynebacterium*
OTU34	527	0	3	0	0	*Bacteria; Proteobacteria; Alphaproteobacteria; Rickettsiales; mitochondria*
OTU35	0	0	7	304	7	*Bacteria; Firmicutes; Bacilli; Bacillales; Paenibacillaceae; Paenibacillus*
OTU36	230	378	184	164	55	*Bacteria; Proteobacteria; Gammaproteobacteria; Pseudomonadales; Moraxellaceae; Acinetobacter; Acinetobacter_rhizosphaerae*
OTU37	0	3	22	499	63	*Bacteria; Firmicutes; Bacilli; Bacillales; Planococcaceae; Sporosarcina*
OTU38	0	0	0	0	20	*Bacteria; Firmicutes; Bacilli; Lactobacillales; Lactobacillaceae; Lactobacillus; Lactobacillus_reuteri*
OTU39	1	181	17	0	15	*Bacteria; Firmicutes; Bacilli; Lactobacillales; Lactobacillaceae; Pediococcus*
OTU40	0	0	21	11	356	*Bacteria; Proteobacteria; Alphaproteobacteria; Rhizobiales; Brucellaceae*
OTU41	328	4	2	0	0	*Bacteria; Cyanobacteria; Chloroplast; Streptophyta*
OTU42	1	88	113	219	167	*Bacteria; Proteobacteria; Betaproteobacteria; Burkholderiales; Alcaligenaceae; Achromobacter*
OTU43	0	167	28	4	30	*Bacteria; Firmicutes; Bacilli; Bacillales; Paenibacillaceae; Paenibacillus*
OTU44	0	0	0	0	194	*Bacteria; Bacteroidetes; Sphingobacteriia; Sphingobacteriales; Sphingobacteriaceae*
OTU45	1	0	0	0	84	*Bacteria; Bacteroidetes; Bacteroidia; Bacteroidales; Bacteroidaceae; Bacteroides*
OTU46	2	0	0	0	111	*Bacteria; Proteobacteria; Gammaproteobacteria; Xanthomonadales; Xanthomonadaceae; Wohlfahrtiimonas*

OTU47	0	0	4	114	0	*Bacteria; Firmicutes; Clostridia; Clostridiales; Clostridiaceae; Alkaliphilus*
OTU48	0	88	77	2	0	*Bacteria; Firmicutes; Clostridia; Clostridiales; Lachnospiraceae*
OTU49	0	0	0	0	216	*Bacteria; Tenericutes; Mollicutes; Acholeplasmatales; Acholeplasmataceae; Acholeplasma*
OTU50	0	0	0	0	173	*Bacteria; Tenericutes; Mollicutes; Acholeplasmatales; Acholeplasmataceae; Acholeplasma*
OTU51	0	0	0	0	15	*Bacteria; Proteobacteria; Alphaproteobacteria; Rhizobiales; Xanthobacteraceae; Xanthobacter*
OTU52	0	0	0	0	226	*Bacteria; Bacteroidetes; Flavobacteriia; Flavobacteriales; Flavobacteriaceae; Myroides*
OTU53	0	0	0	0	239	*Bacteria; Actinobacteria; Actinobacteria; Actinomycetales*
OTU54	0	0	0	99	10	*Bacteria; Firmicutes; Bacilli; Bacillales; Paenibacillaceae; Paenibacillus*
OTU55	9	67	6	25	0	*Bacteria; Proteobacteria; Gammaproteobacteria; Pseudomonadales; Pseudomonadaceae; Pseudomonas; Pseudomonas_nitroreducens*
OTU56	0	53	10	0	0	*Bacteria; Firmicutes; Clostridia; Clostridiales; Clostridiaceae; Clostridium; Clostridium_butyricum*
OTU57	0	0	8	133	0	*Bacteria; Firmicutes; Clostridia; Clostridiales; Tissierellaceae; Tissierella_Soehngenia*
OTU58	0	7	49	106	57	*Bacteria; Proteobacteria; Alphaproteobacteria; Rhizobiales; Brucellaceae; Ochrobactrum*
OTU60	0	0	3	53	190	*Bacteria; Firmicutes; Bacilli; Bacillales; Planococcaceae; Lysinibacillus*
OTU61	0	0	16	63	0	*Bacteria; Firmicutes; Clostridia; Clostridiales; Tissierellaceae; Tissierella_Soehngenia*
OTU62	0	0	0	0	92	*Bacteria; Firmicutes; Erysipelotrichi; Erysipelotrichales; Erysipelotrichaceae; Erysipelothrix*
OTU63	0	0	0	0	126	*Bacteria; Bacteroidetes; Bacteroidia; Bacteroidales; Porphyromonadaceae; Dysgonomonas*
OTU64	54	1	16	0	0	*Bacteria*
OTU65	1	1	0	49	1	*Bacteria; Firmicutes; Bacilli; Bacillales; Bacillaceae*
OTU66	0	0	0	30	0	*Bacteria; Firmicutes; Clostridia; Clostridiales*
OTU67	26	11	84	34	0	*Bacteria; Proteobacteria; Alphaproteobacteria; Sphingomonadales; Sphingomonadaceae; Novosphingobium*
OTU68	0	0	0	0	70	*Bacteria; Actinobacteria; Actinobacteria; Actinomycetales; Micrococcaceae*
OTU69	0	0	0	34	0	*Bacteria; Firmicutes; Bacilli; Bacillales; Paenibacillaceae; Paenibacillus*
OTU70	0	0	6	81	83	*Bacteria; Firmicutes; Bacilli; Bacillales; Paenibacillaceae; Paenibacillus*
OTU71	1	14	42	53	73	*Bacteria; Proteobacteria; Gammaproteobacteria; Xanthomonadales; Xanthomonadaceae; Stenotrophomonas; Stenotrophomonas_acidaminiphila*
OTU72	84	5	3	1	0	*Bacteria; Proteobacteria; Gammaproteobacteria; Pseudomonadales; Pseudomonadaceae; Pseudomonas*

OTU73	13266	1966	4086	791	10	*Bacteria; Proteobacteria; Gammaproteobacteria; Enterobacteriales; Enterobacteriaceae*
OTU74	0	0	19	40	87	*Bacteria; Firmicutes; Bacilli; Bacillales; Paenibacillaceae; Paenibacillus*
OTU75	0	0	0	21	0	*Bacteria; Firmicutes; Clostridia; Clostridiales; Lachnospiraceae*
OTU76	0	0	0	0	93	*Bacteria; Firmicutes; Bacilli; Lactobacillales; Aerococcaceae; Facklamia*
OTU77	0	0	0	23	2	*Bacteria; Firmicutes; Clostridia; Clostridiales; Lachnospiraceae*
OTU78	0	0	0	0	35	*Bacteria*
OTU79	0	0	0	0	47	*Bacteria; Tenericutes; Mollicutes; Acholeplasmatales; Acholeplasmataceae; Acholeplasma*
OTU80	0	0	0	0	36	*Bacteria; Proteobacteria; Betaproteobacteria; Burkholderiales; Alcaligenaceae; Oligella*
OTU81	0	0	0	0	94	*Bacteria; Bacteroidetes; Sphingobacteriia; Sphingobacteriales; Sphingobacteriaceae*
OTU82	0	2	1	0	2	*Bacteria; Firmicutes; Bacilli; Lactobacillales*
OTU83	0	0	0	0	46	*Bacteria; Firmicutes; Bacilli; Bacillales; Paenibacillaceae*
OTU84	0	0	0	0	38	*Bacteria; Bacteroidetes; Bacteroidia; Bacteroidales; Porphyromonadaceae; Parabacteroides*
OTU86	0	0	0	0	2	*Bacteria; Proteobacteria; Gammaproteobacteria; Pasteurellales*
OTU87	0	0	1	53	0	*Bacteria; Firmicutes; Clostridia; Clostridiales*
OTU89	1	0	0	0	1	*Bacteria; Proteobacteria; Gammaproteobacteria; Xanthomonadales; Xanthomonadaceae*
OTU90	0	0	1	21	0	*Bacteria; Proteobacteria; Gammaproteobacteria; Pseudomonadales; Pseudomonadaceae*
OTU91	1	3	7	29	30	*Bacteria; Proteobacteria; Gammaproteobacteria; Enterobacteriales; Enterobacteriaceae*
OTU92	0	5	0	0	28	*Bacteria; Bacteroidetes; Flavobacteriia; Flavobacteriales; Weeksellaceae; Wautersiella*
OTU93	0	0	0	0	17	*Bacteria; Bacteroidetes; Flavobacteriia; Flavobacteriales; Flavobacteriaceae; Myroides; Myroides_odoratimimus*
OTU94	0	0	0	0	26	*Bacteria; Actinobacteria; Actinobacteria; Actinomycetales; Microbacteriaceae; Leucobacter*
OTU95	15	307	727	390	6015	*Bacteria; Bacteroidetes; Sphingobacteriia; Sphingobacteriales; Sphingobacteriaceae; Sphingobacterium; Sphingobacterium_multivorum*
OTU96	1	11	0	44	8	*Bacteria; Firmicutes; Bacilli; Bacillales; Paenibacillaceae; Paenibacillus*
OTU97	0	0	0	0	12	*Bacteria; Bacteroidetes; Flavobacteriia; Flavobacteriales; Weeksellaceae; Wautersiella*
OTU98	1	8	8	0	0	*Bacteria; Firmicutes; Bacilli; Bacillales; Paenibacillaceae; Paenibacillus*
OTU101	0	10	0	0	2	*Bacteria; Bacteroidetes; Flavobacteriia; Flavobacteriales; Weeksellaceae; Wautersiella*

OTU102	0	11	0	0	0	*Bacteria*
OTU103	0	0	7	12	44	*Bacteria; Firmicutes; Bacilli; Bacillales; Paenibacillaceae; Paenibacillus*
OTU104	0	0	0	0	26	*Bacteria; Bacteroidetes; Sphingobacteriia; Sphingobacteriales; Sphingobacteriaceae; Sphingobacterium*
OTU105	0	0	0	0	1	*Bacteria; Actinobacteria; Actinobacteria; Actinomycetales; Dietziaceae; Dietzia*
OTU106	0	0	5	4	4	*Bacteria; Proteobacteria; Alphaproteobacteria; Rhizobiales; Brucellaceae*
OTU107	0	0	7	0	0	*Bacteria; Proteobacteria; Betaproteobacteria; Burkholderiales; Comamonadaceae; Verminephrobacter*
OTU108	0	0	0	0	24	*Bacteria; Proteobacteria; Gammaproteobacteria*
OTU109	0	3	4	3	448	*Bacteria; Firmicutes; Bacilli; Lactobacillales; Enterococcaceae*
OTU110	0	0	3	16	2	*Bacteria; Firmicutes; Bacilli; Bacillales*
OTU111	0	1	4	13	16	*Bacteria; Proteobacteria; Alphaproteobacteria; Rhizobiales; Brucellaceae*
OTU112	0	0	0	0	7	*Bacteria; Actinobacteria; Actinobacteria; Actinomycetales; Brevibacteriaceae; Brevibacterium*
OTU114	0	0	0	0	2	*Bacteria; Firmicutes; Erysipelotrichi; Erysipelotrichales; Erysipelotrichaceae; Erysipelothrix*
OTU115	72	49	92	78	0	*Bacteria; Proteobacteria; Gammaproteobacteria; Enterobacteriales; Enterobacteriaceae*
OTU116	293	134	210	171	0	*Bacteria; Proteobacteria; Gammaproteobacteria*
OTU117	0	3	5	0	0	*Bacteria; Proteobacteria*
OTU118	9	2	1	4	0	*Bacteria; Proteobacteria; Betaproteobacteria; Burkholderiales; Oxalobacteraceae*
OTU119	0	0	6	12	102	*Bacteria; Firmicutes; Bacilli; Bacillales*
OTU120	8	0	2	0	0	*Bacteria; Proteobacteria; Betaproteobacteria; Neisseriales; Neisseriaceae; Vitreoscilla*
OTU122	76	36	42	33	0	*Bacteria; Proteobacteria; Gammaproteobacteria*
OTU123	6	0	0	0	0	*Bacteria; Proteobacteria; Alphaproteobacteria; Rickettsiales; mitochondria*
OTU124	528	1121	1457	1055	1	*Bacteria; Proteobacteria; Gammaproteobacteria; Xanthomonadales; Xanthomonadaceae; Stenotrophomonas*
OTU125	0	0	0	0	12	*Bacteria; Proteobacteria; Epsilonproteobacteria; Campylobacterales; Campylobacteraceae; Arcobacter*
OTU126	105	137	194	74	4447	*Bacteria; Proteobacteria; Gammaproteobacteria; Pseudomonadales; Moraxellaceae; Acinetobacter*
OTU127	8	0	0	0	0	*Bacteria; Proteobacteria; Alphaproteobacteria; Rhizobiales; Bartonellaceae*
OTU129	0	3	33	23	603	*Bacteria; Firmicutes; Bacilli; Bacillales; Planococcaceae*

OTU130	24	28	51	27	0	*Bacteria; Proteobacteria; Gammaproteobacteria; Xanthomonadales; Xanthomonadaceae; Stenotrophomonas*
OTU131	1	1	0	0	11	*Bacteria; Proteobacteria; Betaproteobacteria; Neisseriales; Neisseriaceae; Vitreoscilla*
OTU132	0	0	0	0	5	*Bacteria; Bacteroidetes; Sphingobacteriia; Sphingobacteriales; Sphingobacteriaceae; Sphingobacterium*
OTU134	541	3015	2594	2015	2	*Bacteria; Proteobacteria; Gammaproteobacteria; Xanthomonadales; Xanthomonadaceae; Stenotrophomonas*
OTU135	3	0	0	0	0	*Bacteria; Firmicutes; Clostridia; Clostridiales; Tissierellaceae*
OTU136	8	7	13	9	4	*Bacteria; Proteobacteria; Gammaproteobacteria; Pseudomonadales; Moraxellaceae; Acinetobacter*
OTU137	0	0	4	1	0	*Bacteria; Firmicutes; Clostridia; Clostridiales; Lachnospiraceae*
OTU138	0	0	0	0	14	*Bacteria; Firmicutes; Clostridia; Clostridiales; Clostridiaceae*
OTU139	0	0	0	3	0	*Bacteria; Firmicutes; Bacilli; Bacillales*
OTU140	0	0	0	0	7	*Bacteria; Proteobacteria; Betaproteobacteria; Burkholderiales*
OTU141	1	2	0	0	0	*Bacteria; Proteobacteria; Betaproteobacteria; Burkholderiales; Oxalobacteraceae*
OTU142	0	0	0	0	10	*Bacteria; Bacteroidetes; Sphingobacteriia; Sphingobacteriales; Sphingobacteriaceae; Sphingobacterium*
OTU143	1	0	3	1	0	*Bacteria; Proteobacteria; Alphaproteobacteria; Rhizobiales; Rhizobiaceae; Agrobacterium*
OTU144	0	0	0	0	24	*Bacteria; Firmicutes; Bacilli; Lactobacillales; Carnobacteriaceae*
OTU146	3	0	0	0	0	*Bacteria; Proteobacteria; Gammaproteobacteria; Xanthomonadales; Xanthomonadaceae*
OTU147	0	0	0	1	170	*Bacteria; Firmicutes; Bacilli; Bacillales; Bacillaceae*
OTU149	0	0	0	0	3	*Bacteria; Firmicutes; Bacilli; Bacillales; Paenibacillaceae*
OTU150	1	0	5	1	0	*Bacteria; Actinobacteria; Actinobacteria; Actinomycetales; Microbacteriaceae*
OTU151	0	3	0	1	9	*Bacteria; Proteobacteria; Betaproteobacteria; Burkholderiales; Comamonadaceae*
OTU152	285	589	160	69	226	*Bacteria; Proteobacteria; Gammaproteobacteria; Pseudomonadales; Moraxellaceae; Acinetobacter*
OTU153	0	0	0	0	6	*Bacteria; Firmicutes; Bacilli; Bacillales; Paenibacillaceae*
OTU154	0	0	0	0	3	*Bacteria; Firmicutes; Bacilli; Bacillales*
OTU155	2	1	2	2	0	*Bacteria; Proteobacteria; Gammaproteobacteria; Xanthomonadales; Xanthomonadaceae; Stenotrophomonas*
OTU156	0	0	0	0	4	*Bacteria; Firmicutes; Bacilli; Bacillales*
OTU157	21	16	21	20	0	*Bacteria; Proteobacteria; Gammaproteobacteria*

OTU158	0	0	2	0	0	*Bacteria; Bacteroidetes; Flavobacteriia; Flavobacteriales; Flavobacteriaceae*
OTU159	0	0	0	0	3	*Bacteria; Bacteroidetes; Sphingobacteriia; Sphingobacteriales; Sphingobacteriaceae*
OTU160	0	0	5	3	16	*Bacteria; Firmicutes; Bacilli; Bacillales; Bacillaceae; Bacillus*
OTU161	0	0	0	0	2	*Bacteria; Firmicutes; Erysipelotrichi; Erysipelotrichales; Erysipelotrichaceae*
OTU162	0	9	14	150	164	*Bacteria; Proteobacteria; Betaproteobacteria; Burkholderiales; Alcaligenaceae*
OTU163	0	1	0	3	0	*Bacteria; Tenericutes; Mollicutes*
OTU164	495	257	561	260	39	*Bacteria; Proteobacteria; Gammaproteobacteria; Enterobacteriales; Enterobacteriaceae; Enterobacter*
OTU165	0	0	0	0	21	*Bacteria; Firmicutes; Bacilli; Bacillales; Planococcaceae*
OTU166	0	1	0	0	3	*Bacteria; Firmicutes; Bacilli; Lactobacillales*
OTU167	0	8	23	67	34	*Bacteria; Bacteroidetes; Flavobacteriia; Flavobacteriales; Flavobacteriaceae; Myroides*
OTU168	0	0	0	0	5	*Bacteria; Firmicutes; Bacilli; Lactobacillales*
OTU170	0	0	0	0	4	*Bacteria; Bacteroidetes; Sphingobacteriia; Sphingobacteriales; Sphingobacteriaceae; Sphingobacterium*
OTU171	0	0	0	0	5	*Bacteria; Firmicutes; Bacilli; Lactobacillales*
OTU172	57	46	64	29	2	*Bacteria; Proteobacteria; Gammaproteobacteria; Enterobacteriales; Enterobacteriaceae*
OTU173	1	0	2	29	0	*Bacteria; Firmicutes; Bacilli; Bacillales; Planococcaceae; Sporosarcina*
OTU174	0	0	0	0	3	*Bacteria; Bacteroidetes; Bacteroidia; Bacteroidales; Porphyromonadaceae; Dysgonomonas*
OTU175	0	0	2	0	3	*Bacteria; Proteobacteria; Betaproteobacteria; Burkholderiales; Alcaligenaceae; Pigmentiphaga*
OTU176	3	8	12	10	0	*Bacteria; Proteobacteria; Gammaproteobacteria; Xanthomonadales; Xanthomonadaceae; Stenotrophomonas*
OTU177	7	2	5	1	2	*Bacteria; Firmicutes; Bacilli; Bacillales; Paenibacillaceae; Ammoniphilus*
OTU178	0	0	0	0	4	*Bacteria; Proteobacteria; Betaproteobacteria; Burkholderiales; Comamonadaceae*
OTU179	0	2	0	0	0	*Bacteria; Proteobacteria; Gammaproteobacteria*
OTU180	0	0	14	11	21	*Bacteria; Proteobacteria; Betaproteobacteria; Burkholderiales; Comamonadaceae*
OTU181	0	0	0	2	0	*Bacteria; Firmicutes; Clostridia; Clostridiales; Lachnospiraceae; Coprococcus*
OTU182	0	0	0	0	6	*Bacteria; Bacteroidetes; Flavobacteriia; Flavobacteriales; Weeksellaceae*
OTU183	32	12	16	13	0	*Bacteria; Proteobacteria; Gammaproteobacteria*

OTU184	40	38	53	34	5	*Bacteria; Proteobacteria; Gammaproteobacteria; Enterobacteriales; Enterobacteriaceae*
OTU185	6	63	45	35	2	*Bacteria; Proteobacteria; Gammaproteobacteria; Pseudomonadales; Pseudomonadaceae*
OTU186	0	0	2	0	0	*Bacteria; Firmicutes; Clostridia; Clostridiales; Lachnospiraceae*
OTU187	0	0	0	0	10	*Bacteria; Actinobacteria; Actinobacteria; Actinomycetales; Microbacteriaceae*
OTU191	0	0	0	0	3	*Bacteria; Bacteroidetes; Sphingobacteriia; Sphingobacteriales; Sphingobacteriaceae*
OTU192	3	0	0	0	0	*Bacteria; Proteobacteria; Alphaproteobacteria; Rickettsiales; mitochondria*
OTU193	0	0	0	24	0	*Bacteria; Firmicutes; Bacilli; Bacillales; Paenibacillaceae; Paenibacillus*
OTU194	0	0	2	0	0	*Bacteria; Proteobacteria; Gammaproteobacteria; Xanthomonadales; Xanthomonadaceae; Stenotrophomonas*
OTU195	0	0	0	0	31	*Bacteria; Firmicutes; Erysipelotrichi; Erysipelotrichales; Erysipelotrichaceae; Erysipelothrix*
OTU196	0	0	1	4	0	*Bacteria; Proteobacteria; Gammaproteobacteria; Enterobacteriales; Enterobacteriaceae*
OTU198	2	0	0	0	0	*Bacteria; Proteobacteria; Alphaproteobacteria; Rhizobiales*
OTU199	0	3	2	33	25	*Bacteria; Proteobacteria; Betaproteobacteria; Burkholderiales; Alcaligenaceae; Alcaligenes; Alcaligenes_faecalis*
OTU200	0	1	0	1	107	*Bacteria; Firmicutes; Bacilli; Lactobacillales*
OTU201	0	0	0	0	5	*Bacteria; Proteobacteria; Alphaproteobacteria; Rhodobacterales; Rhodobacteraceae; Paracoccus*
OTU202	0	0	0	0	3	*Bacteria; Firmicutes; Clostridia; Clostridiales; Tissierellaceae; Helcococcus*
OTU203	0	0	0	0	2	*Bacteria; Bacteroidetes; Bacteroidia; Bacteroidales; Porphyromonadaceae; Paludibacter*
OTU204	5	6	3	5	0	*Bacteria; Proteobacteria; Gammaproteobacteria; Pseudomonadales; Pseudomonadaceae*
OTU205	0	0	0	0	3	*Bacteria; Actinobacteria; Actinobacteria; Actinomycetales; Microbacteriaceae; Microbacterium*
OTU206	0	1	0	5	0	*Bacteria; Bacteroidetes; Bacteroidia; Bacteroidales; Prevotellaceae; Prevotella; Prevotella_copri*
OTU207	0	0	0	0	2	*Bacteria; Firmicutes; Bacilli*
OTU208	0	0	0	17	0	*Bacteria; Firmicutes; Bacilli; Bacillales; Planococcaceae*
OTU209	0	0	0	0	4	*Bacteria; Actinobacteria; Actinobacteria; Actinomycetales; Microbacteriaceae*
OTU210	0	0	0	0	57	*Bacteria; Firmicutes; Bacilli; Bacillales*
OTU211	0	0	0	0	10	*Bacteria; Bacteroidetes; Sphingobacteriia; Sphingobacteriales; Sphingobacteriaceae*
OTU212	0	0	2	0	0	*Bacteria; Firmicutes; Clostridia; Clostridiales; Lachnospiraceae*

OTU213	3	0	0	0	0	*Bacteria; Proteobacteria*
OTU214	0	0	3	0	0	*Bacteria; Firmicutes; Clostridia; Clostridiales; Eubacteriaceae; Garciella*
OTU215	0	0	0	0	3	*Bacteria; Bacteroidetes; Bacteroidia; Bacteroidales; Bacteroidaceae; Bacteroides; Bacteroides_ovatus*
OTU217	0	0	0	0	4	*Bacteria; Bacteroidetes; Flavobacteriia; Flavobacteriales; Flavobacteriaceae; Flavobacterium; Flavobacterium_gelidilacus*
OTU218	0	4	1	0	0	*Bacteria; Proteobacteria; Gammaproteobacteria; Xanthomonadales; Xanthomonadaceae*
OTU219	1	0	0	0	0	*Bacteria; Proteobacteria; Alphaproteobacteria; Rhizobiales*
OTU220	10	0	0	0	0	*Bacteria; Proteobacteria; Alphaproteobacteria; Rickettsiales; mitochondria*
OTU222	0	1	0	0	3	*Bacteria; Firmicutes; Bacilli; Lactobacillales; Lactobacillaceae; Lactobacillus; Lactobacillus_brevis*
OTU223	1	1	2	2	1	*Bacteria; Proteobacteria; Alphaproteobacteria; Sphingomonadales; Sphingomonadaceae; Kaistobacter*
OTU224	1	0	0	1	0	*Bacteria; Proteobacteria; Betaproteobacteria; Burkholderiales; Comamonadaceae; Limnohabitans*
OTU225	0	0	3	9	1	*Bacteria; Firmicutes; Bacilli; Bacillales; Bacillaceae*
OTU226	0	0	2	16	1	*Bacteria; Proteobacteria; Gammaproteobacteria; Enterobacteriales; Enterobacteriaceae*
OTU228	123	266	141	71	261	*Bacteria; Proteobacteria; Gammaproteobacteria; Pseudomonadales; Moraxellaceae; Acinetobacter*
OTU229	0	0	0	3	0	*Bacteria; Firmicutes; Bacilli; Bacillales; Bacillaceae*
OTU231	6454	1626	6447	2636	90	*Bacteria; Proteobacteria; Gammaproteobacteria; Enterobacteriales; Enterobacteriaceae*
OTU233	0	0	0	0	1	*Bacteria; Actinobacteria; Actinobacteria; Actinomycetales; Actinomycetaceae; Actinomyces*
OTU236	2	15	17	4	62	*Bacteria; Bacteroidetes; Sphingobacteriia; Sphingobacteriales; Sphingobacteriaceae; Sphingobacterium; Sphingobacterium_multivorum*
OTU237	23	38	18	3	1	*Bacteria; Proteobacteria; Gammaproteobacteria; Pseudomonadales; Moraxellaceae; Acinetobacter*
OTU238	0	0	0	0	2	*Bacteria; Proteobacteria; Gammaproteobacteria*
OTU240	0	0	0	0	2	*Bacteria; Bacteroidetes; Bacteroidia; Bacteroidales; Bacteroidaceae; Bacteroides*
OTU241	6	0	0	0	0	*Bacteria; Proteobacteria; Alphaproteobacteria; Rickettsiales; mitochondria*
OTU243	2	0	2	21	0	*Bacteria; Firmicutes; Bacilli; Bacillales; Bacillaceae*
OTU244	0	0	0	0	10	*Bacteria; Firmicutes; Bacilli; Lactobacillales*
OTU245	0	0	1	1	98	*Bacteria; Firmicutes; Bacilli; Bacillales*
OTU246	0	8	4	5	0	*Bacteria; Proteobacteria; Gammaproteobacteria; Xanthomonadales; Xanthomonadaceae; Stenotrophomonas*

OTU247	0	0	0	0	5	*Bacteria; Bacteroidetes; Bacteroidia; Bacteroidales; Porphyromonadaceae; Dysgonomonas*
OTU248	0	0	0	0	2	*Bacteria; Bacteroidetes; Saprospirae; Saprospirales; Saprospiraceae*
OTU249	106	57	62	60	60	*Bacteria; Proteobacteria; Gammaproteobacteria; Pseudomonadales; Moraxellaceae; Acinetobacter*
OTU250	13377	5049	4270	3811	38	*Bacteria; Proteobacteria; Gammaproteobacteria*
OTU253	46	68	19	22	0	*Bacteria; Proteobacteria; Gammaproteobacteria; Pseudomonadales; Pseudomonadaceae*
OTU255	0	0	0	2	3	*Bacteria; Bacteroidetes; Sphingobacteriia; Sphingobacteriales; Sphingobacteriaceae; Sphingobacterium; Sphingobacterium_multivorum*
OTU256	0	0	0	0	2	*Bacteria; Verrucomicrobia; Verrucomicrobiae; Verrucomicrobiales; Verrucomicrobiaceae; Akkermansia; Akkermansia_muciniphila*
OTU257	1	136	95	662	4233	*Bacteria; Proteobacteria; Betaproteobacteria; Burkholderiales; Comamonadaceae*
OTU258	0	0	0	2	6	*Bacteria; Firmicutes; Bacilli; Bacillales; Bacillaceae*

WB24h, WB48h, WB72h and WB96h refer to moistened wheat bran not treated with housefly larvae after 24, 48, 72 and 96 h. WBMd96h refers to moistened wheat bran treated with housefly larvae for 96 h. Each treatment included three biological replicates.

Supplementary Table S3. The IDs of shared and unique OTUs in the Venn diagram (Fig. 3) of the WB96h and WBMd96h samples.

Group	Shared OTU number/Unique OTU number	OTU ID
WB96h-vs-WBMd96h	78	Otu1,Otu103,Otu106,Otu109,Otu11,Otu110,Otu111,Otu119,Otu124,Otu126,Otu129,Otu13,Otu134,Otu136,Otu14,Otu147,Otu15,Otu151,Otu152,Otu16,Otu160,Otu162,Otu164,Otu167,Otu17,Otu172,Otu177,Otu180,Otu184,Otu185,Otu19,Otu199,Otu20,Otu200,Otu21,Otu22,Otu223,Otu225,Otu226,Otu228,Otu23,Otu231,Otu236,Otu237,Otu24,Otu245,Otu249,Otu25,Otu250,Otu255,Otu257,Otu258,Otu26,Otu3,Otu30,Otu35,Otu36,Otu37,Otu4,Otu40,Otu42,Otu43,Otu5,Otu54,Otu58,Otu6,Otu60,Otu65,Otu7,Otu70,Otu71,Otu73,Otu74,Otu77,Otu8,Otu91,Otu95,Otu96
WB96h	38	Otu115,Otu116,Otu118,Otu122,Otu130,Otu137,Otu139,Otu143,Otu150,Otu155,Otu157,Otu163,Otu173,Otu176,Otu181,Otu183,Otu193,Otu196,Otu204,Otu206,Otu208,Otu224,Otu229,Otu243,Otu246,Otu253,Otu47,Otu48,Otu55,Otu57,Otu61,Otu66,Otu67,Otu69,Otu72,Otu75,Otu87,Otu90
WBMd96h	87	Otu10,Otu101,Otu104,Otu105,Otu108,Otu112,Otu114,Otu12,Otu125,Otu131,Otu132,Otu138,Otu140,Otu142,Otu144,Otu149,Otu153,Otu154,Otu156,Otu159,Otu161,Otu165,Otu166,Otu168,Otu170,Otu171,Otu174,Otu175,Otu178,Otu182,Otu187,Otu191,Otu195,Otu197,Otu2,Otu201,Otu202,Otu203,Otu205,Otu207,Otu209,Otu210,Otu211,Otu215,Otu217,Otu222,Otu233,Otu238,Otu240,Otu244,Otu247,Otu248,Otu256,Otu27,Otu28,Otu29,Otu31,Otu32,Otu33,Otu38,Otu39,Otu44,Otu45,Otu46,Otu49,Otu50,Otu51,Otu52,Otu53,Otu62,Otu63,Otu68,Otu76,Otu78,Otu79,Otu80,Otu81,Otu82,Otu83,Otu84,Otu86,Otu89,Otu9,Otu92,Otu93,Otu94,Otu97

The OTU IDs are identical to those in Supplementary Table S1-2. WB96h refers to moistened wheat bran not treated with house fly larvae after 96 h. WBMd96h refers to moistened wheat bran treated with house fly larvae for 96 h. Each treatment included three biological replicates.
